# Factors influencing the professional identity of nursing interns: a cross-sectional study

**DOI:** 10.1186/s12912-022-00983-2

**Published:** 2022-07-25

**Authors:** Lihong Zeng, Qirong Chen, Sisi Fan, Qifeng Yi, Wenhong An, Huan Liu, Wei Hua, Rong Huang, Hui Huang

**Affiliations:** 1 Xiangya School of Nursing, Central South University, Changsha, Hunan China; 2 Hunan Labor and Human Resources Vocational College, Changsha, Hunan China; 3 The Third Xiangya Hospital, Central South University, Changsha, Hunan China; 4 School of Health and Wellness, Panzhihua University, Chengdu, Sichuan China

**Keywords:** Nursing interns, Clinical internship, Professional identity

## Abstract

**Background:**

Improving the professional identity of nursing intern is significant for enhancing the number of new registered nurses and easing the shortage of nursing personnel. The clinical internship is a key period for the formulation of professional identity. However, we know little about the factors influencing the nursing interns’ professional identity during clinical internship. Therefore, this study explore the influencing factors of nursing interns’ professional identity during clinical internship. This study will provide evidence and suggestions for generating effective strategies contributing to professional identity improvement of nursing interns.

**Methods:**

This was a cross-sectional study. The convenience sampling was used to recruit 398 nursing interns from a teaching hospital in Hunan, China. The demographic characteristics information was collected by a self-developed questionnaire. The nursing interns’ professional identity and potential influencing factors (e.g., work atmosphere, teacher capacity) were measured by questionnaires with good psychometric properties. The appropriate indicators were used for descriptive statistics, and *t test*, analysis of variance, Pearson’s correlation analysis and multiple linear regression were used to analyse the influencing factors.

**Results:**

In this study, the influencing factors of nursing interns' professional identity are education level, first choice of major, residential status, work atmosphere, and teacher capacity. The results showed that: (1) the nursing interns with a higher education level reported a lower level of professional identity; (2) the nursing interns whose first choice of major was not nursing discipline reported a lower level of professional identity; (3) the nursing interns live in rural areas (compared to urban areas) reported a higher level of professional identity; (4) the nursing interns in better work atmosphere reported a higher level of professional identity; (5) the nursing interns under the guidance of the teachers equipped with better teaching capacity reported a higher level of professional identity.

**Conclusion:**

The education level, first choice of major and residential status are influence factors of nursing interns’ professional identity. The nursing educators need to pay attention to nursing interns whose first choice is not nursing, and in a bachelor program, who may have a lower level of professional identity. It is crucial to enhance the nursing interns’ professional identity by improve the work atmosphere and clinical teachers’ capacity, to promote nursing interns to choose nursing as a profession and reduce the shortage of nursing workforce.

## Background

The high turnover rate and the shortage of nurses have become a severe problem worldwide. Nurse turnover rates range from 12 to 60% [1–3], and similarly, the turnover rates for nurses who have been on the job for only 1 year range from 8 to 69% [4–6]. In addition, the WHO estimates that the world is facing a shortfall of 9 million nurses and midwives needed to achieve and sustain universal health coverage by 2030 [7]. The high turnover rate of new nurses not only aggravates the shortage of nursing human resources but also affects the development of the nursing profession [8]. Improving nursing interns’ intention of choosing nursing as a career after graduation is important for alleviating the shortage of nursing personnel. Studies have shown that professional identity is an important factor influencing nursing interns’ career choices [9, 10], i.e., a lower professional identity among nursing interns results in a greater intention to quit [2, 11], which leads to a loss of nursing talents.

For nursing students, professional identity refers to the process of career planning and confirming their professional role in their current status [8]. The nursing students' professional identity formedin practice is closely related to their future career choice [12]. The professional identity of nursing students will affect their transition from student nursing to professional nursing and their willingness to remain in the nursing profession [13]. As nursing students enter the field of nursing, their professional identity serves as the foundation of their nursing practice and affects their entire career [14]. A previous study concluded that nursing students with a lower level of professional identity are more likely to quit, while nursing students with a strong professional identity are more motivated to work in nursing [15] and show a higher retention rate [10]. Therefore, it is important to explore the professional identity of nursing students.

Clinical internship refers to developing clinical competence [16] and conducting professional activities during the last year of undergraduate under the supervision of clinical teacher in teaching hospitals, the time last 12 months [17]. The clinical internship is part of clinical education. The nursing intern is the nursing student in clinical internship. Clinical internship is a key period for nursing students to form professional identity [2]. Some studies have shown that during clinical internship, nursing students compare imagined practice with reality by becoming familiar with the work environment, and finally forming an objective professional identity [18, 19]. Some research showed that the role of clinical workers in treating the patient, the force of example from practice mentors, clinical professionals’ attitude to nursing students, relationships, and negative experiences have an important impact on nursing students during the clinical internship [2, 20]. But there have few studies to verified if those factors can influence the nursing interns’ professional identity.

Recently, many studies focus on the influencing factors of the professional identity of nursing student in college. The influence factors of professional identity among the nursing students in university are as follows: Firstly, demographic characteristics such as the gender (the female nursing students’ professional identity is higher) [21], education level (the diploma students’ professional identity is higher than baccalaureate nursing students) [12]. Secondly, the power of model, such as role modelling [13] and the model of teacher [22] could improve students’ professional identity. Lastly, the students with higher self-efficacy and resilience have a higher level of professional identity [23]. However, there is a paucity of studies focusing on the influencing factors of nursing interns’ professional identity during clinical internship.

Therefore, this study aims to explore the status and influencing factors of the professional identity of nursing interns. This study will provide evidence and suggestions for generating effective strategies contributing to improve the nursing interns’ professional identity during clinical internship.

## Method

### Study setting

This study was conducted at a public hospital in Changsha of Hunan province, China. The area of Changsha is 11,819.5 km^2^. In 2021, the population estimated with 10,239,3000 in Changsha. The annual growth rate is 4.42% based on the 2021 population enumeration. Total of 32 tertiary hospitals in Changsha. All the tertiary hospitals have the qualification to conduct the clinical internship. We selected the Third Xiangya Hospital of Central South University to conduct this research. The Third Xiangya Hospital is a tertiary hospital, and combines health, research and teaching. The Third Xiangya Hospital included more than 1990 beds and 800 clinical nurses, and approximately 350–450 nursing students for internship per year. This teaching hospital accepts nursing interns who completed theoretical courses in different nursing schools.

### Study design

A cross-sectional study was conducted to explore the factors influencing nursing interns’ professional identity during clinical internship. This study was performed followed by the STROBE checklist.

### Participants

The sample size of this study was calculated according to the sample size calculation principle used in Kendall’s cross-sectional investigation, i.e., *n* = independent variable × (5–10). The independent variables included demographic information, the Professional Identification Scale score (three subscales) and the Nursing Clinical Learning Environment Evaluation Scale score (six subscales). Considering a 20% of invalid questionnaire rate, a minimum final sample size of 192 cases was required.

Convenience sampling was used to select 438 nursing interns from a teaching hospital in Hunan Province (The interns were managed by the teaching hospital once the clinical internship was started). The nursing interns from this teaching hospital were recruited at the end time of their internships. The inclusion criteria of participants were as follows: 1) full-time junior, college, and undergraduate nursing students who practiced in the hospital and 2) completed the internship.

### Data collection

Data were collected from January to May 2019 in the teaching hospital. After the interns completed the internship, the researchers distributed questionnaires (paper version) to the participants face to face. The questionnaire did not require the participant to provide the identified information (Such as name, intern ID). After the participant completed the questionnaire, the researcher checked the questionnaire immediately. If there is any omission, the participant will be reminded to fill in the missing items in time, we did not require the participant to fill the missing items if the participant refuse to complement. All participants complete the questionnaires in a quiet environment (an office in the ward). All of the data were kept in a locked cupboard and on a password-protected computer to ensure confidentiality, and only viewed by researchers in our study group.

### Instruments

#### Demographic characteristics questionnaire

The demographic characteristics questionnaire was generated by the researcher and consisted of questions concerning gender, age, education level, only child in family, residential status, first major choice is nursing or not (In China, students can select more than one career choice after completing the entrance examination. If their scores of the entrance exam cannot meet the requirement of their first choices, they are transferred, with consent, to another major, although it may not be their preferred major), academic grade in school, school level.

#### Professional identification scale

This scale was developed by Brown [24] in 1986. It consisted of three subscales (professional cognition – three items, professional impact – three items, and professional evaluation – four items) including a total of 10 items, with the responses to each item provided on a 5-point Likert scale (1 = never to 5 = very often). The total score of the scale ranged from 10 to 50. A higher average score on the scale indicates a stronger professional identity. The scoring standard of each dimension was the same as the total average score. The Cronbach’s α for the total scale was 0.71. Hong Lu [25] translated the Professional Identification Scale into Chinese through a combination of the Brislin model, back-translating instruments and committee approach, and the Cronbach’s α of the Chinese version was 0.82. In this study, the Cronbach’s α of the scale was 0.84. The author permits us to use the Professional Identification Scale .

#### Environment evaluation scale for clinical nursing internship

The scale was developed by Chinese scholar Hu Laxian in 2011. The scale includes a total of 30 items. This scale was divided into six subscales (teaching method, teacher capacity, learning opportunities, interpersonal relationships, working atmosphere and organizational support), and every subscale contained five items. The responses to each item are provided on a 5-point Likert scale (1 = never to 5 = very often). The total score of the scale ranged from 30 to 150. A higher score indicates a higher evaluation of the clinical learning environment. The Cronbach’s α of this scale was 0.96, and the cumulative contribution rate of the six common factors was 73.37%. The correlation coefficients between the dimensions ranged from 0.48 to 0.66. The correlation coefficients between each subscale and the scale ranged from 0.83 to 0.86. The set validity correlation coefficients ranged from 0.56 to 0.84, while the correlation coefficients of discriminant validity ranged from 0 to 0.46 [26]. In this study, the Cronbach’s α of each dimension ranged from 0.72 to 0.95, and the overall Cronbach’s α of the scale was 0.96. The author permits us to use the Environment Evaluation Scale for clinical internship.

### Data analysis

SPSS version 22.0 was used for data analysis. Frequency and percentage are used to describe ordinal and categorized variables, such as gender, education, and first major choice. The means and standard deviations are used to describe continuous variables whose data are in accordance with normal distribution, such as age, professional identity, and clinical learning environment. The scores on the Professional Identification Scale, Nursing Clinical Learning Environment Evaluation Scale and each subscale closely approached normal distribution. The *t test* was used to evaluate if there is a statistically significant difference in professional identity scores between the two groups that were divided by gender, education, first major choice, and residential status. Analysis of variance (ANOVA) was used to analyse the differences in professional identity scores between three or more groups, that were divided by academic grade in school and school level. Pearson’s correlation was applied to explore the correlation between the nursing interns’ professional identity and the dimensions of the clinical learning environment evaluation. The influencing factors of professional identity were analysed via multiple linear regression. Statistical significance for all analyses was set at *P* < 0.05.

### Ethical considerations

Ethical approval was obtained from the Institutional Review Committee of The Third Xiangya Hospital of Central South University (No: 2020-S003). Before the data collection, all participants were informed of the purpose and procedures of this study. Both verbal and written explanations of the survey and the purpose of the research were provided to the participants. The information sheet was distributed before participation, and written informed consent was obtained before the completion of the questionnaire. Participation in the study was voluntary. Interns had the right to withdraw from the survey at any time. All the information collected has been maintained in strict confidence.

Confidentiality and anonymity were maintained by not asking for names and by numbering the questionnaires, with each participant receiving a number on a debriefing sheet. Any student who wanted to withdraw his or her data prior to the commencement of data analysis could do so by contacting the researcher. All data were kept in a locked cupboard and on a password-protected computer to ensure privacy.

## Results

### Demographic characteristics of the participants

A total of 438 questionnaires were collected, for a return rate of 100%. However, because 40 questionnaires had incomplete data, the valid questionnaire rate is 90.87%. Finally, a total of 398 nursing interns provided a valid questionnaire in this study. The characteristics of the participants and the relationship between the demographic variables and the professional identity total score are presented in Table 1. There were 208 nursing interns at the diploma level and 190 at the baccalaureate level. Among all participants, 26.38% identified as being the only child in their families, and the proportion of nursing interns living in rural areas (46.98%) was close to the proportion of nursing interns living in urban areas (53.02%). Nursing interns whose first major choice is nursing accounted for only 30.65% of all participants.

**Table 1:** Demographic characteristics and single factor analysis of Professional Identity in clinic nursing students (*N* = 389)

Variables	*n (%)/Mean ± SD*	Professional Identity		
		*Mean ± SD*	*t/F*	*P*
Professional identity	4.02 ± 0.63			
Gender				
Male	38 (9.55)	4.02 ± 0.10	0.30	0.58
Female	360 (90.45)	4.02 ± 0.03		
Education level				
Diploma level	208 (52.26)	4.16 ± 0.04	5.23	0.02
Baccalaureate level	190 (47.74)	3.87 ± 0.05		
Only child in family				
Yes	105 (26.38)	3.88 ± 0.07	7.85	0.01
No	293 (73.62)	4.07 ± 0.03		
Residential status				
Rural	187 (46.98)	4.16 ± 0.04	10.60	0.00
Urban	211 (53.02)	3.87 ± 0.05		
First major choice				
Yes	276 (69.35)	4.15 ± 0.04	4.44	0.04
No	122 (30.65)	3.73 ± 0.06		
Academic grade in school				
Excellent	197 (49.50)	4.08 ± 0.04	2.02	0.13
Medium	188 (47.24)	3.97 ± 0.05		
Poor	13 (3.26)	3.82 ± 0.16		
School level				
Junior college	208 (52.26)	4.16 ± 0.04	15.35	0.00
Second level university	92 (23.12)	4.01 ± 0.06		
First level university	98 (24.62)	3.74 ± 0.07		

### The factors influencing professional identity of the participants

#### Demographic factors influencing professional identity

The nursing interns’ professional identity was at a medium level (4.02 ± 0.63) (Table 1). The professional identity score among nursing interns who were the not only child in their families (4.07 ± 0.03) was higher than that among nursing interns who were the only child (3.88 ± 0.07, *P*<0.05). The professional identity score of nursing interns from rural areas (4.16 ± 0.04) was higher than that of nursing interns from urban areas (3.87 ± 0.05, *P*<0.01). The professional identity score of nursing interns whose first major choice is nursing (4.15 ± 0.04) was higher than that of nursing interns whose first major choice is not nursing (3.73 ± 0.06, *P*<0.05). The professional identity score of nursing interns with a college diploma (4.16 ± 0.04) was higher than that of nursing interns with a baccalaureate diploma (3.87 ± 0.05), and the difference was significant (*P*<0.05). The professional identity of nursing interns in junior college (4.16 ± 0.04) exhibited the highest score, followed by second-level university (4.01 ± 0.06) and first-level university (3.74 ± 0.07) interns, and there were significant differences (*P*<0.01) (Table 1).

#### Correlation between the clinical learning environment and professional identity of nursing interns

The correlation analysis results of each factor are shown in Table 2, the results show that the professional identity of nursing interns was positively correlated with teaching methods (*r* = 0.34) and interpersonal relationships (*r* = 0.39, *P* < 0.01). Professional identity was also positively correlated with teacher capacity (*r* = 0.40), learning opportunities in the clinical learning environment (*r* = 0.35) and work atmosphere (*r* = 0.41, *P* < 0.01). In addition, there was a positive correlation between professional identity and organizational support (*r* = 0.30).

**Table 2:** Correlation between nursing students’ professional identity and dimensions of the environment evaluation scale for clinical nursing internship

	Professional identity	Teaching method	Teacher capacity	Learning opportunities	Interpersonal relationships	Working atmosphere	Organizational support
Professional identity	1	0.34**	0.40**	0.35**	0.39**	0.41**	0.30**
Teaching method		1	0.77**	0.71**	0.61**	0.68**	0.62**
Teacher capacity			1	0.70**	0.73**	0.76**	0.62**
Learning opportunities				1	0.73**	0.74**	0.72**
Interpersonal relationships					1	0.85**	0.70**
Working atmosphere						1	0.72**
Organizational support							1

#### A multiple linear regression of influencing factors on the professional identity of nursing interns

To ensure a stable model, education, only child in family, residential status, first major choice, school level, teaching method, teacher capacity, learning opportunities, interpersonal relationship, work atmosphere, organizational support and clinical learning were included in a multiple linear regression based on the results of the *t test*, ANOVA and correlation analysis. A multiple linear regression provided the degree of variance in nursing interns’ professional identity that could be accounted for by influencing factors. In Table 3, the results showed that education level (*β =* − 0.12, *P*<0.05) and first choice of major (*β =* − 0.23, *P*<0.01), residential status (*β =* 0.12, *P*<0.05), work atmosphere (*β =* 0.22, *P*<0.01) and teacher capacity (*β =* 0.17, *P*<0.05) were independently associated with the professional identity of nursing interns in a clinical environment, and 26% of the variance was explained.

**Table 3:** Results of the multiple linear regression analysis for predicting nursing students’ professional identity

Variables	*B*	*S.E.*	*β*	*t*	*p*
Constant	2.65	0.27		9.73	0.00
Demographic characteristics					
Residential status	0.15	0.06	0.12	2.72	0.01
Education	−0.15	0.06	−0.12	−2.60	0.01
First major choice	−0.31	0.06	−0.23	−5.05	0.00
Nursing clinical learning environment					
Working atmosphere	0.04	0.01	0.22	3.31	0.00
Teacher capacity	0.04	0.01	0.17	2.52	0.01

*R* = 0.52, *R*^*2*^ = 0.27, *Adjusted R*^*2*^ = 0.26, *P* < 0.001

## Discussion

We did not find recent research on the professional identity of nursing interns abroad. Most international studies focus more on clinical nurses’ professional identity [27–30]. However, the exploration of the professional identity of nursing interns is also crucial for alleviating nurse shortage and the development of nursing as a profession and a discipline.

In the analysis of demographic characteristics, the professional identity of nursing interns from rural areas was markedly higher than that of interns from urban areas. The reason may be that rural areas have a lower economic level and poorer working conditions [21]. The nursing interns from low-income areas are inclined to consider the income and the stability work [31]. Accordingly, this finding suggests that the professional identity of nursing interns is related to the environment where they grew up. In addition, the stronger professional identity of nursing interns whose first major choice is nursing, because of their more expect and interest in nursing [32]. Therefore, the will of nursing interns should be respected in the choice of major, as it can influence nursing intern whether to choose nurse as their career. For interns whose first choice is not nursing, the educator should change their attitudes toward nursing and reinforce their professional identity by creating a positive internship experience [13]. The results also indicated that the nursing interns’ professional identity related to education level, and the professional identity of undergraduate nursing interns with a lower level of professional identity. This may be the nurses have a relatively low social status and are not respected by most of the public [33]. However, nursing interns with a higher educational level have higher social expectations and hope to obtain a higher social status [21]. Thus, the nation, colleges and nursing educators should set a positive example and public the importance of nurses by media.

This study also reported that work atmosphere and teacher capacity were independent factors affecting nursing interns’ professional identity. In a good work atmosphere, the nurses have a positive attitude to work, and provide a high-quality service to the patient [9]. Those nurses establish a model for nursing interns [33]. The role of model is very important to form the professional identity [33]. The teacher’s capacity was also an important factor affecting nursing interns’ professional identity. Research showed that professional identity is closely related to confidence [34]. The clinical teachers have the stronger abilities, the nursing interns are more likely to have excellent competence [35]. The competence contributes to the nursing interns’ confidence [36], which benefits to cultivate their professional identity. Therefore, to improve the professional identity of nursing interns, the teaching hospitals should not only create a good work atmosphere but also improve the capacity of the clinical teachers who are responsible for guiding the nursing interns.

However, in this study, the difference of professional identity between the male interns and female interns was not statistically significant. The reason may be that this study did not include enough sample of male interns for identifying the difference. In future studies, the researcher could recruit more male nursing internships. Furthermore, the results of this study suggested that the teaching method, learning opportunity and organizational support also were not the influencing factors of nursing interns’ professional identity. It is different from a previous study [37]. Those factors belong to nursing training [38]. Research reported that the higher level of professional identity related to nursing training [39]. However, there have heavy burden of clinical work for nursing interns in China, may be the nursing interns do not get enough training, which leads to a lower level of professional identity. A study reported that interpersonal relationships is important to professional identity [40]. But in this study, the interpersonal relationship does no effect on nursing interns’ professional identity. May be during the COVID-19, the students live with family, the interpersonal relationship will be more harmonious compared to clinical internship [41]. Although the gender, teaching method, learning opportunity, organizational support and interpersonal relationship did not influence the nursing interns’ professional identity, the nursing educators still take those factors seriously.

Therefore, there are many strategies could be considered to improve the nursing interns’ professional identity in a clinical learning environment. At the national level, laws and regulations should be formulated to ensure the legitimate rights and interests of nurses are being enforced and efforts should be made to improve their social status [42]. In addition, positive images of nurses should be established through media, films and so on, which will not only enhance the social identity and professional identity of the nursing profession but also promote pride in the profession [41]. From the perspective of teaching hospitals, hospitals should focus on the work atmosphere and clinical teachers’ capacity in the clinical environment [2]. Clinical teachers should pay more attention to the interns with lower professional identity and provide them with sufficient practice chances to gain positive clinical experience [13]. Furthermore, nursing education managers can provide individualized practice plans for nursing interns and training programs for clinical teachers. In addition, we should also provide a supportive practice environment and offer timely communications with nursing interns. These changes could make nurses feel more respected and will improve the practice experience of nursing interns [43, 44].

### Limitations

This study used a convenient sample and just recruitment participants in a tertiary teaching hospital in Hunan, China. So, the representativeness of sample size needed to improve. Nursing still has a lower status compared with other careers in China. Therefore, in different contexts, there may have different factors affecting on professional identity of nursing interns, the influenced factors in different contexts need to be further explored. In addition, the study was limited to a descriptive cross-sectional study, the casual relationship cannot be answered. The instrument can be got the permission before the study. The strategies proposed in the discussion require further empirical studies to evaluate their effectiveness. Next step, we will explore some multi-dimensional interventions to improve professional identity of nursing interns and take targeted measures to intervene the nursing interns.

## Conclusion

The nursing interns’ professional identity is related to some demographic characteristics (i.e., residential status, first major choice, education). So, the educator should pay attention to the nursing interns who lived in the urban, not first choice of nursing and with a high education level. Except, the working atmosphere and teacher ability have effect on the nursing interns’ professional identity. The teaching hospital needs to improve the working atmosphere and teacher capacity by some measures. Nursing educators should pay more attention to enhancing nursing interns’ practical ability and increasing the willingness of nursing interns to join the nursing team to alleviate the nurse shortage worldwide. More empirical studies are needed to further explore the effective strategies for improving the professional identity of nursing interns.

## Data Availability

The datasets generated and analysed during the current study are not publicly available because this work is part of a larger study. The datasets are available from the corresponding author upon reasonable request.
